# Natural History of Isolated Skull Fractures in Children

**DOI:** 10.7759/cureus.3078

**Published:** 2018-07-31

**Authors:** Saif Hassan, Abdul Q Alarhayema, Stephen M Cohn, John C Wiersch, Mitchell R Price

**Affiliations:** 1 Surgery, St. Luke's the Woodlands Hospital, Woodland, USA; 2 Surgery, University of Texas Health Science Center, Texas, USA; 3 Surgery, Staten Island University Hospital, Queens Village, USA; 4 Surgery, University of Texas, Texas, USA; 5 Pediatric Surgery, Northwell Health at Staten Island University Hospital, Staten Island, USA

**Keywords:** emergency department (ed), glasgow coma scale (gcs), intracranial lesions (icl), national trauma database (ntdb), skull fracture (sf), traumatic brain injury (tbi).

## Abstract

Head injury is the most common cause of neurologic disability and mortality in children. We had hypothesized that in children with isolated skull fractures (SFs) and a normal neurological examination on presentation, the risk of neurosurgical intervention is very low. We retrospectively reviewed the medical records of all children aged six to sixteen years presenting to our Level 1 trauma center with traumatic brain injuries between January 1, 2006 and December 31, 2014. We also analyzed the National Trauma Data Bank (NTDB) research data set for the years 2012-2014 using the same metrics. During this study period, our center admitted 575 children with skull fractures, 197 of which were isolated (no associated intracranial lesions (ICLs)). Of the 197 patients with isolated SFs, 155 had a normal neurological examination at presentation. In these patients, there were no fatalities and only three (1.9%) required surgery, all for the elevation of the depressed skull fracture. Analyzing the NTDB yielded similar results. In 5,194 children with isolated SFs and a normal neurological examination on presentation, there were no fatalities and 249 (4.8%) required neurosurgical intervention, almost all involving craniotomy/craniectomy and/or elevation of the SF segments. In conclusion, children with non-depressed isolated skull fractures and a normal Glasgow coma scale (GCS) at the time of initial presentation are at extremely low risk of death or needing neurosurgical intervention.

## Introduction

Head injury remains the number one cause of neurologic disability and death in children. Every year, traumatic brain injury (TBI) in children results in an estimated 2,685 deaths, 37,000 hospitalizations, and 600,000 emergency department (ED) visits [1-2]. Fortunately, more than 98% of these head injuries are classified as mild, and less than 0.1% of the children need to undergo neurosurgical intervention. Skull fractures account for the majority of head injury-related hospitalizations. Recent studies have also reported admission rates for isolated skull fractures to be as high as 78%, yet more than 85% are discharged within one day of admission [3]. Pediatric traumatic brain injury associated hospitalizations account for more than one billion dollars in total charges annually [2]. In patients with isolated skull fractures, hospital costs are more than triple in patients hospitalized compared to those discharged from the emergency department [3]. These data lead us to question the need for hospitalization in children with skull fractures with minimal associated brain injury [3].

There is a growing body of evidence suggesting that neurologically normal children with isolated skull fractures can be safely discharged from the ED without hospitalization, and endure no increased risk of adverse clinical outcomes [4]. The purpose of this study was to examine management strategies and clinical outcomes in children presenting with skull fractures at our Level 1 trauma center and nationally. We hypothesized that in children with isolated skull fractures and a normal neurological examination at the time of presentation, the risk of death from neurological deterioration was low.

## Materials and methods

We retrospectively reviewed the medical records of all children aged six to sixteen years presenting to our Level 1 trauma center with traumatic brain injuries between January 1, 2006 and December 31, 2014. We also analyzed the National Trauma Data Bank (NTDB) research dataset between 2012–2014 using the same metrics.

Only children with isolated skull fractures identified on head computed tomography (CT) scans were considered for analysis. Isolated skull fracture was defined as displaced or non-displaced skull fractures without evidence of intracranial lacerations/hemorrhage. Both entities were selected using the International Classification of Diseases, Ninth Revision, Clinical Modification (ICD-9-CM) codes. In analyzing the NTDB, patients with traumatic brain injuries and Abbreviated Injury Scale (AIS) scores greater than three in body regions other than the head were excluded from the analysis.

Our primary outcomes were traumatic brain injury (TBI) related mortality and performance of neurosurgical procedures. Secondary outcomes included hospital and intensive care unit (ICU) length of stay in patients who survived to discharge. Data recorded included demographic information, injury mechanism, admission vital signs and neurological exam, injury severity score (ISS), the incidence of neurosurgical interventions, and clinical outcomes.

Neurosurgical interventions included craniotomy or craniectomy, ventricular drainage procedures, elevation of depressed skull fractures, and/or closure of meningeal defects.

The study protocol was reviewed and approved by the University of Texas Health Science Center at the San Antonio Institutional Review Board and the University Health System Medical Dental Research Committee. SPSS software, version 22 (Statistical Package for Social Sciences, Chicago, IL, USA) was used for all analyses.

A p-value of < 0.05 was regarded as statistically significant. Continuous numerical variables were analyzed by a two-sample t-test or one-way analysis of variance (one-way ANOVA). Categorical variables were analyzed by the χ2 test or Fischer’s exact test. All numerical data were expressed as the mean ± standard deviation (SD).

## Results

During the study period, we admitted 575 children with skull fractures diagnosed on head CT, of whom 197 had isolated skull fractures and 378 had associated intracranial lesions (ICLs). 

In patients with isolated skull fractures, 155 (78.7%) presented with a normal neurological examination (Glasgow coma scale (GCS) 15).  In this cohort, there were no fatalities, and three (1.9%) required a repair or elevation of the depressed skull fracture segments greater than 1 cm in depth. Eight patients (5.2%) were discharged directly from the ED, and the overall median hospital length of stay (LOS) was two days.

There were 42 patients that presented with isolated non-basilar skull fractures and a depressed mental status (GCS < 15). Eleven of these patients had basilar skull fractures, and none had a depression greater than 10 mm. In this cohort, there were no deaths and no neurosurgical intervention was performed. The median hospital length of stay (LOS) was three days, with 71.4% being admitted to the ICU (Table 1).

**Table 1:** Outcomes in children with isolated skull fractures at a Level 1 trauma center Clinical outcomes in children with skull fractures at our Level 1 trauma center. In the patients with isolated non-depressed skull fractures, there were no fatalities and only three patients underwent neurosurgical interventions, all for elevation of depressed skull fracture segments. GCS: Glasgow coma scale LOS: Length of stay ICU: Intensive care unit

	Isolated skull fractures at a Level 1 trauma center	
	GCS 15	GCS < 15
Mortality	0/155 (0%)	0/42 (0%)
Neurosurgical procedure	3/155 (1.9%)	0/42 (0%)
Hospital LOS (median)	2 days	3 days
ICU LOS (median)	0 days	2 days

We identified 378 patients with skull fractures associated with intracranial lesions (ICLs), of which there were 32 TBI-related deaths.

Analysis of the NTBD dataset

In analyzing the NTBD after excluding children with an AIS score greater than three in regions other than the head, we identified 6,647 children aged six through sixteen years with isolated skull fractures.

In children with isolated skull fractures, 5,194 (78.1%) presented with a normal neurological examination (GCS 15). In this group, there were five fatalities, all of which were children with basilar skull fractures. Overall, 249 children (4.8%) required neurosurgical intervention. The most frequent procedures were the elevation of depressed skull fractures (50.2%, 125/249) and craniotomy/craniectomy with or without the evacuation of hematoma or repair of meninges (47.0%, 117/249). Overall, 119 patients were discharged from the ED and the median hospital length of stay (LOS) for the group was two days.

There were 1,543 patients that presented with isolated skull fractures and a depressed mental status (GCS < 15). In this cohort, there were 162 deaths (11.4%) and 238 patients (15.4%) required neurosurgical intervention: 38.2% were for intracranial pressure monitoring, 19.3% involved elevation of depressed skull fractures, while 42.4% were craniotomy/craniectomy. The median hospital length of stay (LOS) was three days and 73.9% of patients were admitted to the ICU (Table 2).

**Table 2:** Outcomes in children with isolated skull fractures using the National Trauma Data Bank dataset 2012-2014 A nationwide analysis of clinical outcomes in children with isolated skull fractures using the National Trauma Databank dataset 2012-2014. Children with a depressed neurological examination on admission (GCS < 15) were much more likely to require a neurosurgical intervention than those with an admission GCS = 15 (15.4% vs. 4.8%, p < 0.05). GCS: Glasgow coma scale LOS: Length of stay ICU: Intensive care unit

	Isolated Skull fractures using the NTDB 2012-2014	
	GCS 15	GCS < 15
Mortality	5/5,194 (0.1%)	162/1,543 (10.5%)
Neurosurgical procedure	249/5,194 (4.8%)	238/1,543 (15.4%)
Hospital LOS (median)	2 days	3 days
ICU LOS (median)	0 days	1 days

## Discussion

The purpose of our study was to investigate whether children with skull fractures without intracranial pathology had a low likelihood of mortality secondary to neurological deterioration, and/or neurosurgical intervention. Our data suggest that while neurologically intact children with isolated skull fractures have an exceedingly low incidence of death (all five fatalities in children with a normal GCS were seen with basilar skull fractures), 4.8% of children required neurosurgical intervention, almost all of which were the elevation of depressed skull fractures. This study is consistent with our previous work in younger children (age birth to five years) with skull fractures [4].

In the US, the rate of hospitalizations for pediatric TBI has decreased tremendously over the last several decades [5]. From 1980 to 1995, the estimated annual incidence rate declined by more than 60% [6]. From 1991 to 2005, the rate further decreased by 39%, settling at 72.7 hospitalizations per 100,000 [5]. Although mediated by improved diagnostic technology, this decline in hospitalizations was largely attributable to changes in hospital practice. Admission rates for moderate and severe traumatic brain injuries remained relatively unchanged over this time period [5], but in children with mild TBI care were shifted to the outpatient setting [6]. During this time period, TBI-related deaths in children significantly declined, with a 22% decrease in mortality between 1979 and 1992, and a 20% decrease from 1991 to 2005 (currently 2.8 deaths per 100,000). This survival benefit is likely due to improvements in both diagnostic and therapeutic strategies.

TBIs in children account for more than 60,000 hospitalizations annually and are estimated to account for more than one billion dollars in total charges annually [7]. Reducing unnecessary admissions in the pediatric population has undoubtedly contributed to improved public health. Shifting the care of patients at low risk of negative health outcomes to the outpatient setting allows resources to be allocated to populations that truly benefit from inpatient care. Identifying children at risk for intracranial injury and subsequent deterioration while limiting unnecessary hospitalization has been the objective of numerous studies.

In a prospective, multicenter study of more than 40,000 children, Kupperman, et al. reported the incidence of clinically important TBI to be 0.9%, with only 0.1% of patients requiring neurosurgical intervention. Children aged two years and older that were asymptomatic (no loss of consciousness, vomiting, or severe headache), had a normal physical exam (normal mental status and no signs of basilar skull fracture) and a non-severe injury mechanism, requiring neither CT-imaging nor neurosurgical intervention, and could be safely discharged from the ED [8-11]. Skull fractures account for up to 20% of outpatient visits for head trauma in children, and their presence is an indicator of a more substantial force to the head, and are frequently associated with intracranial lesions. The presence of intracranial hemorrhage in patients with skull fractures, however, is a poor prognosticator. The elevation of depressed skull fractures is typically pursued if the depressed segment is more than five mm below the inner table of adjacent bone, while craniotomy/craniectomy is typically done for gross contamination, dural tear with pneumocephalus, underlying hematoma, and severe brain edema.

Our pediatric trauma center receives injured children from a catchment area of approximately 52,000 sq mi (approximately the size of the state of New York). It is thus not unusual that patients and their families are transferred from emergency departments hundreds of miles away for the management of isolated skull fractures due to the lack of neurosurgical capabilities at remote care centers. Some authors have argued that transferring and/or admitting these children is not only unnecessary but also potentially harmful, as the children are caused to spend time away from their families and loved ones while being exposed to the risks of long-distance road/air transport and hospital-borne infections [12]. 

Others, like Mannix et al., found that while 78% of children were hospitalized, almost all were discharged within two days, with only one child requiring a neurosurgical procedure (repair of the cerebral meninges). Hospital costs were more than triple for hospitalized patients compared with patients discharged from the ED ($2,064 versus $619) [3].

The inherent liability of the discharge of the pediatric skull fracture patient must be balanced against the same lack of access due to time-distance factors previously described.

In the urban setting, it is not unreasonable that children with uncomplicated skull fractures be safely discharged home from the ED in the presence of reliable social support. Whether or not such an approach is applicable in a rural setting, where formalized systems of trauma care may be lacking, and delays in care are expected, remains to be determined. 

## Conclusions

Our results indicated that children with non-depressed, isolated skull fractures and a normal GCS score at the time of initial presentation are at very low risk of neurological death. In addition, only approximately 5% of these patients require neurosurgical intervention.
